# The British Medical Association and the Voluntary Hospitals

**Published:** 1922-07

**Authors:** P. R. Cooper

**Affiliations:** Honorary Surgeon, Altrincham Hospital


### CORRESPONDENCE.

[Correspondence on all subjects is invited, but we cannot in any way be responsible for the opinions expressed by our correspondents, who must give name and address as a guarantee of good faith, but not necessarily for publication. Correspondents are reminded that conciseness greatly facilitates early insertion.]

### The British Medical Association and the Voluntary Hospitals.

*To the Editor of* THE HOSPITAL AND HEALTH REVIEW.

SIR,—As one who is deeply interested in the future of our Voluntary Hospitals, may I be allowed to state my views?

(1) *The Definition of a Voluntary Hospital.*—Like yourself, sir, I cannot accept the B.M.A. definition, nor does Mr. Bishop Harman's explanation content me. They make "voluntary management" the sole criterion, waiving aside what are to my mind the two more important criteria—viz., support largely if not entirely by voluntary contributions, and gratuitous service by the medical and surgical staff in all suitable (*i.e.*, generally necessitous) cases. Not one of these three criteria will stand alone as a satisfactory definition, especially if, as appears to be the intention, the two latter criteria are excluded. The objections to their inclusion, however, will not bear examination. Surely neither the fact that a patient in a voluntary hospital is in a position to contribute towards his maintenance, and is asked to do so, nor the fact that a hospital patient can well afford to pay for treatment, and is expected to do so, vitiate the essentially voluntary character of the great bulk of the work in that hospital, which is certainly primarily intended for and mainly used by those who cannot afford to pay for such treatment. Any such payments are, of course, quite voluntary—*i.e.*, not legally enforceable. Again, the fact that an honorary surgeon or physician, who gives his professional services freely and without question to all necessitous cases which come under his care at a hospital, accepts a fee from such patients as are *not* necessitous but well able to pay for those services, by no means cancels the generally charitable character of the bulk of the work at that hospital. (Unless, of course, through gross abuse in admission, the bulk of patients in that hospital are well able to pay for their treatment.)

Finally, in these days of insurance, co-operation, profit-sharing, etc., where large contributions may be made by individual workers, their employers, public companies, and even Government departments, towards the cost of hospital treatment, there is surely nothing unethical, either medically or socially, in the medical men who carry out this treatment receiving some part of these contributions as payment for their services, which, indeed, cannot any longer be regarded as for "charitable objects."

Some verbal purists will contend that this is "contrary to the principle of a voluntary hospital." Perhaps they will state what principle this would contravene. I venture to suggest that voluntary hospitals were not founded on any specific principle—ethical or economic—they have "just growed." They were a human need, and, like every human need, they tend to effect themselves. There is no doubt that during the last 100 years the medical and surgical needs of many thousands of sufferers have been on the whole very effectively met by our voluntary hospitals, nor is that good work rendered less estimable by the fact that, like the "quality of mercy," medical charity has been "twice blessed," not only blessing those who receive its benefits, but those who bestow them, for, incidentally, medical science and practice have also benefited exceedingly, thereby making for the more effectual satisfaction of the needs. But human needs change with the varying conditions of human life—"new occasions teach new duties," etc., and human institutions, if they are to continue effective, must adapt themselves to the altered circumstances. The development of medical and surgical science within the last 50, or even 20 years, the growth of bacteriology, hematology, parasitology, radiology, cardiology and innumerable specialist departments of medicine and surgery, have not only greatly extended our diagnostic and therapeutic powers, but they have vastly increased the expense of medical work and multiplied the need for institutional treatment. Moreover, large numbers of people who could well enough afford the medical expenses of a generation ago are now quite unable to bear the huge costs of modern medical treatment which many of them need. If, however, they can bear a proportion of the cost, or if by some co-operative arrangement or system of insurance, the costs can be reduced to a minimum, there is nothing ethically or economically unsound in permitting this, whilst the gain is all on the side of more efficient treatment and diminished suffering. It becomes, then, a mere quibble or hair-splitting if we refuse to regard a hospital which adapts itself to modern requirements, and allows a certain number of paying or part-paying patients to its wards, as still, on the whole, a "voluntary hospital." If we seek to distinguish such a hospital from a State hospital, we may, if we like, add to our definition of voluntary hospital as here outlined the words "such hospital being neither owned nor controlled by the State."

(2) Perhaps the most important point to be decided is the question of suitability of patients for admission. This may mean (*a*) inability to pay for home treatment, including nursing, etc.; (*b*) on medical or surgical grounds the patient can be better treated—*i.e.*, has a better chance—if in a modern hospital. Under (*a*) there is no question of "suitability for hospital" if the case is one requiring bed treatment, special nursing, etc. Under (*b*) there is much to be said from the medical aspect of the case for admitting these cases to hospital, but some important considerations arise: firstly, there is not hospital accommodation for a tithe of the cases which on these grounds might be considered "suitable," hence it is necessary to give precedence to the really necessitous cases that cannot possibly be attended and nursed at home or in a nursing home. Secondly, there must always be many border-line cases—*i.e.*

cases in which it is difficult to decide, either on medical or financial grounds, or both, as to the suitability of a particular case for hospital treatment. Whilst allowing, however, for the widest latitude and even generosity in deciding in particular cases, it must be admitted that nowadays more and more people who can well afford to pay reasonable fees for medical and surgical treatment are using, or rather, abusing, our present voluntary hospital system, and the only way to check this, it seems to me, is for the hospital almoner to inquire into the means of the patients, and where they can obviously afford to pay a charge should be made. At the same time it must always be remembered that the hospital is primarily intended for necessitous patients, and a large preponderance of beds must always be kept for such patients.

(3) As regards " recommends " for hospital by the patient's own doctor, these will, of course, merely deal with the suitability for admission on medical or surgical grounds, and it seems to me a reasonable and proper procedure. You seem to fear, sir, that it gives the patient's doctor a power of veto on his admission to a hospital. I don't see why this should be so. If a patient wishes to go into hospital, and his doctor refuses to give him a recommend, he can always *change his doctor*, or ask for a consultation with one of the honoraries of the hospital whom he desires to attend him, and if the case is considered suitable there will rarely be any difficulty about the matter. In all cases the question of suitability for admission to a hospital ultimately rests with the honorary staff of that hospital. As a matter of fact, at our hospital we have rarely had any trouble with the patients' doctors, who as a rule are only too glad to get their bad cases into hospital, and are rather inclined to object when for some reason we are unable to admit a case.

(4) The allegation that if some paying or part-paying patients are admitted to a hospital the benefactions of the public will cease, or at any rate be seriously diminished, is one which I cannot accept, at least as long as the essentially voluntary character of the hospital such as I have outlined is maintained. For surely the subscribing public know well enough that there are still a vast number of necessitous cases to be provided for, and that medical and surgical equipment on a grand scale must be maintained, and that this needs more and more money. The falling off in subscriptions has not, I venture to say, been due to this alleged cause, but to the general impoverishment due to the war. It is rather the falling off of income and the greatly increased expenditure which is leading to a general tightening of the conditions for free treatment, which have certainly been far too lax in the past. The great majority of medical men, both connected with hospitals and not, are, I feel sure, in favour of continuing the voluntary hospital system, extended to meet existing needs, rather than submitting to State hospitals run and staffed by State officials.

Yours, etc.,

P. R. Cooper, M.D., F.R.C.S.
Honorary Surgeon, Altrincham Hospital.

### A Hospital's Water Supply.

*To the Editor of* The Hospital and Health Review.

